# Semi-supine Exercise Stress Echocardiography in Children and Adolescents: Feasibility and Safety

**DOI:** 10.1007/s00246-014-1058-4

**Published:** 2014-11-20

**Authors:** P. Ciliberti, I. McLeod, F. Cairello, J. P. Kaski, M. Fenton, A. Giardini, J. Marek

**Affiliations:** 1 Great Ormond Street Hospital for Children, London, WC1N 3JH UK; 2 Institute of Cardiovascular Sciences, University College London, London, UK

**Keywords:** Echocardiography, Exercise test, Pediatric cardiology, Coronary artery disease, Hypertrophic cardiomyopathy

## Abstract

Although exercise stress echocardiography (ESE) is a well-validated technique in adult population, its use in children is quite limited. We aimed to assess the feasibility, the safety and the reproducibility of ESE, using on-line scanning in semi-supine cyclo-ergometer protocol in a large pediatric population. Between July 2008 and January 2013, 42 patients (mean age 14 ± 3) were evaluated with a bicycle ESE performing 50 studies. ESE was successfully performed and well tolerated by all patients. None of the patients presented with adverse effects of stress-induced ischemia. HR was 82 ± 13 at rest, and 153 ± 19.1 during peak exercise. Among 544 views analyzed for grading of image quality, the visualization was optimal in 473 (87 %), suboptimal in 39, and inadequate in 32 (6 %). 37 tests were performed in patients with congenital or acquired coronary abnormality. Regional wall motion abnormalities (RWMA) were revealed in nine cases (24 %). The agreement between the two different observers showed a *K* index of 0.7276 (95 % CI 0.6497–0.8055) for the image quality and a *K* index of 0.5125 (95 % CI 0.4782–0.5468) for the RWMA analysis. Among ten patients with hypertrophic cardiomyopathy, we were able to demonstrate the new comparison of significant left ventricular outflow tract gradient (≥30 mmHg) during exercise in three patients (30 %). Bicycle stress echocardiography performed by on-line scanning during exercise is a feasible, safe, and reproducible modality in children. Further data to assess its diagnostic accuracy are, however, needed. Stress echocardiography provides a dynamic assessment of the myocardial structure and function under conditions of physiologic or pharmacologic stress.

## Background

Stress-induced abnormalities of ventricular wall motion in patients with ischemic heart disease were recognized in 1979 [25]. Since then, stress echocardiography has been extensively applied in patients with coronary artery disease (CAD), and it has become a well-accepted modality for diagnosis, risk stratification, and prognostication in this setting [13, 39]. Its use has subsequently been extended to other clinical settings such as patients with hypertrophic [32, 40, 43] or dilated cardiomyopathy, valvar heart disease [7, 44, 45],pulmonary hypertension [8, 21, 22] and recently for selecting potential responders to cardiac resynchronization therapy [33, 42] and for the selection of donor hearts for cardiac transplantation [6].

The stressor agent can be pharmacologic or physiologic. Dobutamine is the drug most widely used, although adenosine and dipyridamole are also commonly adopted. Physical exercise is the ideal stressor, being able also to provide information about symptoms, exercise capacity, and hemodynamic response to exertion [17, 39]. Exercise stress echocardiography (ESE) is most commonly performed on the treadmill, using similar protocols adopted for exercise ECG, or with a bicycle in either upright or supine position.

Despite wide experience in adults, stress echocardiography in the pediatric age group is rarely used [27, 41] and primarily reserved for patients with Kawasaki disease [28, 37] and pediatric transplant recipients [12, 30]. A small number of reports have been published in patients with congenital heart diseases [11, 23].

The major disadvantage of ESE in pediatric age is the precipitous drop in heart rate after peak exercise compared to adults [37]. In addition, some concerns exist about the image quality due to the higher heart rate in children, and the method’s reproducibility in this setting has not previously been assessed. Bicycle exercise in the semi-supine position, compared to treadmill, offers the advantage that images can be obtained during all stages of exercise (including peak) in real time, avoiding the rapid heart rate drop. Instead of missing the peak heart rate due to its drop from erect position to a bed for imaging, recumbent position and continuous scanning should potentially allow better detection of ischemia and time for higher quality of images. Our aim hence was to assess the feasibility, safety, and reproducibility of dynamic ESE using “on-line” scanning in semi-supine cyclo-ergometer protocol in a wide spectrum of children with different types of disease.

## Methods

### Patients

Data of all patients undergoing a bicycle exercise echocardiography at the echocardiography unit of the Great Ormond Street Hospital for Children between July 2008 and January 2013 were retrospectively analyzed.

### Exercise Echocardiography Protocol

All examinations were performed according to our standardized institutional laboratory protocol. A symptom-limited exercise test was conducted on a semi-supine bicycle ergometer (Lode Medical Technology, Groningen, The Netherlands) using a continuous incremental bicycle protocol with a work rate increment between 5 and 20 W/min according to gender and weight. All patients were given the opportunity to familiarize themselves with the cycle ergometer, and the procedure was discussed with the patient and parent/guardian prior to the test starting. Blood pressure measurement and clinical review preceded all tests. To be able to perform the test, all the children were at least 120 cm in height and above 7 years of age.

Echocardiography images were assessed at baseline, at low work load exercise at peak exercise, and during recovery, using a GE VIVID 7(GE Healthcare, Milwaukee, WI, with 4 × 4 Stress protocol adapted to our protocol). Peak exercise was defined as the maximum level of performance achieved before patient`s exhaustion. The low work load images were acquired after an increase in heart rate by 20 %, during a level of workout that the patient was still comfortable with. All scans were performed with harmonic fusion imaging using a M4S probe. For the assessment of CAD, according to our clinical laboratory protocol, images of the left ventricle (LV) at each exercise stage were obtained from apical 4-chamber, 2-chamber, parasternal long-axis view, and parasternal short-axis view at the levels papillary muscles. All images were recorded digitally and analyzed off-line by the same observer (JM) using a 16-segments, 5-point scale model of the left ventricle according to the recommendations of the European Association of Echocardiography [29].

Studies performed for reasons other than assessment of CAD did not follow this 16-segment protocol at each stage, and instead, a more focused assessment was performed in line with the reason for referral. Specifically, for patients with HCM, an assessment of the presence and extent of systolic anterior motion of the mitral valve (SAM), presence and severity of mitral regurgitation, and left ventricular outflow tract gradient was performed, according to previously published techniques in adults [43].

The image quality was assessed for each acquired view on a three-point scale: 1. complete endocardial definition and wall thickening; 2. inadequate visualization of one or two LV segments; 3. inadequate visualization of three or more LV segments

### Coronary Angiography and Cardiac MRI

When coronary angiography or cardiac MRI was performed within 6 months from the ESE (without any significant clinical event during this period), these exams were used for comparison with the ESE results.

### Interobserver Variability

A representative sample (29, 78 %) of the scans performed for evaluation of coronary artery disease was independently and blindly reviewed off-line by two different observers (PC and IM), not aware of patients’ medical history, for assessment of image quality and presence of regional wall motion abnormalities (RWMA). For the RWMA analysis, the agreement between the two observers was tested for each of the 16 segments at every stage of the protocol. For the image quality, the agreement was tested for each acquired view.

### Statistical Analysis

All continuous variables were presented as mean with standard deviation. Categorical variables were reported as number and percentage. The weighted Cohen’s Kappa test was used to assess the agreement between the two observers. A *K* value <0.20 was considered to reflect poor reproducibility, 0.21–0.40 fair, 0.41–0.60 moderate, 0.61–0.80 good, and 0.81–1.00 was considered suggestive of a very good reproducibility [2].

## Results

### Patients

Forty-two (42) patients (23 males, 55 %, mean age 14 ± 3 years, median age 14.5, range 6–22) were enrolled in the study, and 50 tests were performed. Six patients underwent two studies, and one patient underwent three studies. No differences were found between different studies performed by the same subject. Thirty-seven (74 %) tests were executed for assessment of coronary artery disease, ten (20 %) tests were for assessing LV outflow tract gradient during exercise in patients affected by hypertrophic cardiomyopathy (HCM), and three tests (1 patient affected by dilated cardiomyopathy, 1 patient affected by pulmonary hypertension, and 1 patient affected by dynamic LV outflow tract obstruction) were accomplished for different reasons. The main demographic data of the subjects performing the 50 tests are summarized in Table 1.

**Table 1:** Main clinical characteristics and results of the entire cohort of test performed

	Total, *n* = 50
Male sex, *n* (%)	28 (56 %)
Age at exam (year)	14 ± 3
BSA	1.4 ± 0.5
Indication, *n* (%)	
Coronary artery disease detection	37 (74 %)
HCM	10 (20 %)
Other	3 (6 %)
Patients on medication, *n* (%)	11 (22 %)
Beta blockers	7 (14 %)
Calcium channel blockers	4 (8 %)
ACE inhibitor	2 (4 %)
Termination due to muscular exhaustion, *n* (%)	50 (100 %)
ECG abnormalities during stress, *n* (%)	5 (10 %)
Arrhythmia, *n* (%)	0 (0 %)
Symptoms	2 (4 %)
Rest HR (bpm)	82 ± 13.6
Peak HR (bpm)	153.4 ± 19.7

### Feasibility and Safety

ESE was successfully performed and well tolerated by all patients. Satisfactory images were obtained in the entire cohort, and no patient was excluded due to the poor image quality of the scan. No significant arrhythmia (sustained atrial or ventricular tachycardia) or complication was detected, and all the studies were interrupted for muscular exhaustion. We recorded only a few isolated ventricular ectopic beats in one patient, one patient reported feeling of dizziness, and another patient presented with chest discomfort. In five patients (10 % of the studies), ST depressions were noted on ECG during exercise in absence of symptoms. Two of these patients were subjects with HCM, the remaining three were subjects with suspected CAD, and only in one case, the ST depression was associated to the presence of RWMA. Resting HR was 82 ± 13, and peak HR was 153 ± 19 (81 ± 15; 159 ± 17 excluding heart transplant patients and patients on beta blockers or calcium channel blockers). The main results of all the tests performed are summarized in Table 2.

**Table 2:** Main clinical characteristics and results of the tests performed for detection of coronary abnormalities

	Total, *n* = 37
Male sex, *n* (%)	22 (59 %)
Age at exam (year)	13.6 ± 3
BSA	1.4 ± 0.5
Indications	
Transition to adult clinic	7 (19 %)
Chest pain/clinical deterioration	7 (19 %)
Follow-up	13 (35 %)
Coronary disease previously found at cath/MRI	10 (27 %)
Diagnosis	
TGS S/P ASO	9 (24 %)
Kawasaki disease	7 (19 %)
Heart transplant	11 (30 %)
Congenital coronary abnormality	9 (24 %)
Other	1 (3 %)
Patient on medication, *n* (%)	2 (6 %)
Beta blockers	0 (0 %)
Calcium channel blockers	0 (0 %)
Ace inhibitor	2 (6 %)
Termination due to muscular exhaustion, *n* (%)	37 (100 %)
ECG abnormalities during stress, *n* (%)	3 (8 %)
Arrhythmia, *n* (%)	0 (0 %)
Symptoms	1 (3 %)
Rest HR (bpm)	87 ± 12.2
Peak HR (bpm)	155.7 ± 17.9
Coronary angiograms available, *n* (%)	20 (54 %)
Cardiac MRI available, *n* (%)	21 (57 %)
Coronary angio or MRI available	26 (70 %)

### Coronary Artery Disease Analysis

Thirty-seven exams (mean age at the time of the exam 13.6 ± 3) were performed for detection of CAD in 29 patients. Nine (24 %) exams were performed in patients who had undergone repair of congenital coronary abnormalities (five patients with anomalous left coronary artery from the pulmonary artery (ALCAPA), two patients with right coronary artery from the left coronary sinus, one patient with left coronary artery from the right coronary sinus with intramural course, one patient with left main coronary artery occlusion of unknown origin), 11 (30 %) in heart transplant recipients, 7 (19 %) in patients with coronary artery abnormalities after Kawasaki disease, and 9 (24 %) in patients who had previously undergone arterial switch operation for transposition of the great arteries. One (3 %) patient was referred for typical chest pain after surgical repair for double outlet right ventricle. The indication for the test was chest pain or clinical deterioration in 7 (19 %) cases, clinical follow-up in 13 (35 %), CAD previously found at coronary angiography or MRI in 10 (27 %) and in 7 (19 %) cases the test was performed as part of the routine protocol in our institution prior to transition to adult services. One patient reported mild dizziness during recovery. Resting HR was 87 ± 12.2, and peak HR was 155.7 ± 17.0 (Table 2).

Nine patients were identified to have RWMA. A complete description of these nine cases and the comparison with coronary angiography and cardiac MRI (when available) are shown in Table 3. In the remaining 28 tests without any evidence of RWMA on ESE, coronary angiography or cardiac MRI when available demonstrated significant coronary stenosis in four cases (14 %).

**Table 3:** Systematic description of the nine cases with evidence of RWMA during ESE

Patient	Diagnosis	RWMA at rest	RWMA at low exercise	RWMA at peak exercise	RWMA at recovery	Coronary stenosis >70 % at cath	LG enhancement at MRI
1	TGA S/P ASO	+	+	–	+	–	–
2	HTx	+	–	–	+	+	/
3	CCA	–	+	+	–	/	/
4	HTx	+	+	–	+	–	/
5	HTx	+	+	+	+	+	+
6	HTx	+	+	+	+	+	+
7	CCA	+	+	+	+	–	–
8	TGA S/P ASO	+	+	+	+	–	–
9	TGA S/P ASO	+	+	+	+	+	–

+positive, −negative, /not performed, *CCA* congenital coronary abnormality, *TGA* transposition of the great arteries, *ASO* arterial switch operation, *HTx* heart transplant

### Hypertrophic Cardiomyopathy Population

Ten patients affected by HCM (mean age 14.6 ± 3.6) were studied. No significant arrhythmia was encountered. The indication for ESE in all cases was the clinical suspicion of provocable LV outflow tract obstruction in the absence of a resting gradient on transthoracic echocardiography. Seven patients (70 %) had exertional chest pain, two (20 %) presented with presyncope on exercise, and one patient (10 %) had increasing exercise-related fatigue. Only one patient reported symptoms during the test (mild chest discomfort). Nine patients (90 %) were on medications (7 on beta blockers, 4 on calcium channel blockers, and 3 on both). Resting HR was 74.1 ± 14.2, and peak HR was 134.3 ± 14.9. Mean resting left ventricular outflow tract (LVOT) gradient was 22.5 ± 11.2, and peak LVOT gradient was 39.8 ± 30.5. LVOT gradient was ≥30 mmHg at rest in two patients (20 %), and 3 new patients (30 %) developed a LVOT gradient ≥30 mmHg during exercise. The main data of ESE in HCM population are summarized in Table 4.

**Table 4:** Main clinical characteristics and results of the tests performed in patients with HCM

	Total, *n* = 10
Male sex, *n* (%)	5 (50 %)
Age at exam (year)	14.6 ± 3.6
BSA	1.3 ± 0.5
Indications	
Exertional chest pain	7 (70 %)
Exertional presyncope	2 (20 %)
Exercise-induced fatigue	1 (10 %)
Medication	9 (90 %)
Beta blocker	7 (77 %)
CCBs	4 (44 %)
Termination due to muscular exhaustion, *n* (%)	10 (100 %)
ECG abnormalities during stress, *n* (%)	2 (20 %)
Arrhythmia, *n* (%)	0 (0 %)
Symptoms	1 (10 %)
Resting HR (bpm)	74.1 ± 14.2
Peak HR (bpm)	134.3 ± 14.9
LVOT gradient at rest, mmHg	22.5 ± 11.2
LVOT gradient at peak exercise, mmHg	39.8 ± 30.5
LVOT gradient at rest ≥30 mmHg, *n* (%)	2 (20 %)
LVOT gradient ≥30 mmHg at peak exercise, *n* (%)	5 (50 %)

*CCBs* calcium channel blockers, *LVOT* left ventricle outflow tract

### Image Quality and Interobserver Variability

Image quality and the interobserver variability were analyzed in 29 patients (1 exam for each patient assessed for potential coronary artery disease). Four hundred and sixty four different views were analyzed. 403 (87 %) views were judged as optimal (score = 1), 32 (7 %) as suboptimal (score = 2), and 29 (6 %) as inadequate (score = 3). The view more inclined to be suboptimal was the two-chamber view for assessing the anterior and inferior LV wall.

The coefficient of agreement (*K*) among the two investigators for the analysis of image quality was 0.7276 (95 % CI 0.6497–0.8055), representing a good correlation. For the wall motion analysis (2329 segments analyzed), the *K* was 0.5125 (95 %CI 0.4782–0.5468), representing a moderate agreement between the two observers.

## Discussion

Stress echocardiography in children is not as well established as a diagnostic modality as it is in the adult population. The scarce data available in this setting, characterized by a wide heterogeneity in terms of characteristics of the studied populations and type of stressor, have very likely contributed to the lack of a shared protocol in the pediatric community. Our study shows that bicycle stress echocardiography performed by on-line scanning during exercise is a feasible, well-tolerated, and reproducible modality in children, and our protocol could be safely applied by other specialists in pediatric echocardiography.

The wide heterogeneity in terms of the studied cohort and indications for testing is testified by the presence of isolated reports on use of stress echocardiography in children in completely different clinical settings. There are few reports focused on assessing the presence of CAD after heart transplantation [12, 15, 16, 28, 30, 46] or after arterial switch operation in patients born with transposition of the great arteries [19, 23] or in subjects with history of Kawasaki disease [20, 24, 34, 47], as well as studies providing dynamic evaluation in patients with congenital left heart disease [1, 5, 14, 35], repaired tetralogy of Fallot [3, 10], repaired complex congenital heart defects [19, 26, 38], and after Fontan operation [9].

The un-standardized nature of this investigation is demonstrated further by the number of different stressor agents used. Dobutamine, adenosine, and dipyridamole have been used as pharmacological stressors, and both treadmill and bicycle exercise testing have been utilized when ESE. This large heterogeneity in terms of the background of the patients and type of stressor has not encouraged the establishment of an accepted stress echocardiography protocol in children, and may, at least in part, explain the conflicting results in terms of sensitivity and specificity obtained by the different studies assessing children with CAD [12, 19, 23, 46].

The rapid drop of heart rate in children just after the peak exercise phase [36] may be a further contributing factor to these conflicting results. The latter has previously been identified as the most important limitation of the technique, with a rapid return to resting heart rate after stopping exercise, which can occur within 1 min in some children. This limitation is particularly important when the standard treadmill exercise protocol is used, forcing the acquisition of the images after the end of the exercise. In our study, the wide difference between HR at rest and peak HR (82 ± 13.6 at rest, 153.4 ± 19.7 at peak; 81.3 ± 15–158.9 ± 17.1 excluding heart transplant and patients on beta blockers or calcium channel blockers) testifies that bicycle ESE allowing on-line scanning may resolve this technical problem in children.

Image quality is a well-known technical limitation in exercise stress echocardiography [12], so that the use of contrast agents has been also proposed in children [27] to improve LV border delineation. In our experience, using a semi-supine bicycle ergometer, the images were obtained in satisfactory quality in the entire cohort of patients, and none of them was excluded due to the poor image quality of the scan. The systematic analysis of all images confirmed very high quality in more than 85 %.

Concerns have also been raised about the safety of the technique in children, with one report outlining a high prevalence of side effects, including hypertension, arrhythmia, and chest pain during dobutamine stress exercise in children [3]. In our cohort, we experienced mild adverse effects in less than 5 % of patients and no significant arrhythmia.

Very few data are available on reliability of stress echocardiography [31]. To our knowledge, interobserver variability of ESE in children has never been assessed. We tested interobserver variability for image quality of the acquired views, demonstrating a good agreement between the two different observers. A slightly worse but anyway moderately good agreement was found in the grading of regional wall motion abnormalities. Our analysis on grading of RWMA was performed comparing each LV segments analyzed for all the views obtained. This extensive and complete analysis very likely imposed a handicap on the result, and the agreement would have likely been better if the analysis had compared just the presence of RWMA. However, this result might suggest that ESE for assessing of RWMA in children is not a straightforward technique, and should be performed by experienced operators who are properly trained.

We have also demonstrated that ESE may be a valuable technique in children with HCM. A significant number of patients without resting LV outflow tract gradients may develop dynamic obstruction with exercise, and detection of an exercise-induced gradient may provide a therapeutic target for the relief of symptoms as well as enabling more reliable monitoring of the response to therapy [4]. To the best of our knowledge, this is the first study assessing the feasibility of ESE in this setting in the pediatric population, and further, larger studies are warranted to optimize the technique and assess its clinical utility.

### Limitations

Our study did not explicitly focus on ESE diagnostic accuracy of detection of coronary stenosis in children. Although the results comparing ESE with coronary angiography and cardiac MRI were presented, the limited number of patients with simultaneous coronary angiography or cardiac MRI did not allow any statistical analysis to be performed. Even the rate of false positive and false negative in our study must be reassessed in a dedicated prospective study about ESE diagnostic effectiveness. However, the latter could be partially explained by the presence of extensive collateral circulation in children, while it is well known that a sizable proportion (more than 30 %) of patients with positive ESE has no significant coronary lesion in the adult population [18]. Nonetheless in a large adult cohort, outcomes of patients with false-positive results were similar to those of patients with true-positive results, suggesting a prognostic value of the ESE even in absence of CAD, and suggesting that patients with false-positive results on ESE should still receive intensive risk factor management and careful clinical follow-up [18].

## Conclusions

Exercise stress echocardiography is a feasible, safe, and reproducible modality in children. Exercise performed on a semi-supine bicycle ergometer allows acquisition of good quality on-line images and avoids the rapid drop of heart rate after exercise in children representing an ideal type of stressor in pediatric population. Further data are needed to assess its diagnostic accuracy in assessing for the presence of CAD, and its possible role in risk and prognostic stratification in the pediatric setting.

In children with HCM, bicycle stress echocardiography can unmask latent LV outflow tract dynamic obstruction during exercise in patients without a resting gradient.
